# Spiritual well-being and “dark” personality traits: Machiavellianism, narcissism, psychopathy, and sadism

**DOI:** 10.3389/fpsyg.2026.1743599

**Published:** 2026-01-22

**Authors:** Ivan Bakushkin, Regina Ershova

**Affiliations:** 1 Department for Missionary Work and Youth Affairs of the Kolomna Diocese, Moscow, Russia; 2 Department of Psychology and Pedagogy, Faculty of Philology, Peoples’ Friendship University of Russia, Moscow, Russia

**Keywords:** Dark Tetrad, machiavellianism, narcissism, psychopathy, sadism, spiritual well-being, existential well-being, religious well-being

## Abstract

**Introduction:**

The study of human well-being and its contributing factors remains a central concern in psychological science. One under-researched area involves the relationship between spiritual well-being—an integrative indicator of health and life satisfaction—and “Dark Personality” traits: Machiavellianism, psychopathy, narcissism, and sadism. No prior studies have explored this relationship within the Russian population, largely due to the absence of a validated instrument for assessing spiritual well-being. The present study attempts to address this empirical void. The aim of the study was to examine the relationship between spiritual well-being and Dark Tetrad traits in a Russian-speaking sample.

**Materials and methods:**

The empirical sample consisted of 959 Russian-speaking participants (761 women and 198 men; M age = 38.6, SD = 14). Spiritual well-being was assessed using the *Spiritual Well-Being Scale*. The Dark Tetrad traits were measured with the *Short Dark Tetrad Questionnaire*. Data analysis was conducted using R version 4.5.1 (R Foundation for Statistical Computing). Descriptive statistics, Spearman’s rank correlation coefficient (*ρ*), as well as univariate and multivariate linear regression analyses were applied.

**Results:**

The correlational analysis supported the hypothesis of a negative association between spiritual well-being and Machiavellianism, psychopathy, narcissism, and sadism. However, univariate and multivariate regression analyses revealed more complex interactions. For the RWB subscale measured by the SWBS, analysis indicated a significant shift in the association with psychopathy: from negative (*β* = −0.44 [95% CI: −0.61; −0.28]) to positive (*β* = 0.38 [95% CI: 0.21; 0.55], *p* < 0.001) after controlling for sadism. Narcissism was demonstrated to be a strong positive predictor of existential well-being and overall spiritual well-being (*β* = 0.32 [95% CI: 0.22; 0.42], *p* < 0.001 and *β* = 0.26 [95% CI: 0.07; 0.46], *p* = 0.009, respectively).

**Conclusion:**

The spiritual well-being is systematically and negatively associated with the common core of dark personality (D-core). Sadism emerges as the most potent negative predictor, underscoring a fundamental incompatibility between pleasure in cruelty and spiritual health. While Psychopathy and Machiavellianism also reflect this harmful core, Narcissism presents a nuanced picture: its negative association is attributable to its shared variance with the D-core, whereas its residual, agentic components can positively support existential and spiritual well-being. These findings highlight that spiritual well-being is particularly sensitive to the underlying motivational core of dark traits, being eroded by configurations centered on callous self-interest but capable of integrating more adaptive, self-assured qualities.

## 1 Introduction

Spiritual well-being is conceptualized by researchers as an innate drive for meaning and purpose, a sense of uniqueness achieved through this pursuit, and an affirmation that life is moving in the right direction toward a meaningful goal (Fourianalistyawati, 2018). Hungelmann et al. (1985) define spiritual well-being as a multidimensional construct encompassing connections with: oneself (self-knowledge, search for meaning in life) (Elkins et al., 1988); others (care, compassion, gratitude); nature (a reverent, respectful attitude) (Bethelmy and Corraliza, 2019); and God (beliefs and profound relationships beyond the human level, such as with the universe, a transcendent reality, a higher power, or God) (Gomez and Watson, 2023).

Ellison conceptualizes spiritual well-being as a distinct dimension of quality of life, defined as an affirmation of life in relationship with God, self, community, and environment that nurtures and celebrates wholeness (Ellison, 1983). Researchers operationalize it through the Spiritual Well-Being Scale (SWBS), with religious and existential subscales capturing perceived relationship with God and sense of life purpose and satisfaction, respectively, (Ellison and Smith, 1991). Spiritual well-being is a dynamic and integral aspect of humanity through which individuals seek meaning, purpose, and transcendence, and engage in relationships with themselves, family, others, community, society, and all that is meaningful or sacred (Ferrell et al., 2018). Within the same framework, religious well-being refers to the perceived quality of one’s relationship with God, whereas existential well-being denotes a sense of meaning, purpose, and life satisfaction; overall spiritual well-being is defined as *a general sense of life purpose and satisfaction together with a sense of well-being in relation to God* (Paloutzian and Kirkpatrick, 1995). According to this framework, spiritual well-being is regarded as an integrative mental health indicator (Peterman et al., 2002; Whitford and Olver, 2012; Seybold and Hill, 2001).

Podolin-Danner et al. (2022) using Multidimensional Inventory of Religious/Spiritual Well-Being (MI-RSWB) define spiritual well-being as a composite of religious and spiritual life that includes hope, forgiveness, sense of meaning, and perceived closeness to the divine; underscoring SWB as a multifaceted, value-laden sense of existential fulfillment and connectedness (Podolin-Danner et al., 2022). Studies on teachers’ spiritual health portray SWB as a resource composed of meaning, connectedness, and faith that supports psychological well-being (Heidari et al., 2022). Different authors vary in how explicitly they reference God, religion, or transcendence, but converge on the idea that spiritual wellbeing is the subjective experience of spirituality as a state of existential and relational flourishing, typically involving: (1) meaning and purpose; (2) inner peace or harmony; (2) connectedness and relationship; (4) value alignment with demonstrable implications for psychological, physical, and organizational outcomes (Dik et al., 2024).

The term *Dark Triad* was introduced by Paulhus and Williams (Paulhus and Williams, 2002), describing a constellation of “negative” personality traits: Machiavellianism, subclinical psychopathy, and subclinical narcissism. The three traits are all characterized by emotional coldness, low agreeableness, tendency toward deception, primary psychopathy, and inflated self-esteem (Egorova and Sitnikova, 2014). Chabrol and colleagues (Chabrol et al., 2009) expanded this framework by incorporating subclinical sadism, which has been identified as a predictor of violent behavior, cruelty, aggressiveness, masochism, and paraphilias (Antonyan, 2020; Paulhus et al., 2021; Soldatova et al., 2020; Derish, 2021).

The dark personality traits, including sadism, has been examined by Book et al. (2016), Johnson et al. (2019), and Paulhus (2014). Chabrol et al. (2009) analyzed the influence of dark traits on social, familial, and delinquent behavior. The Dark Tetrad has also been studied in relation to social media behavior (Craker and March, 2016; Moor and Anderson, 2019), life cycle and demographic variables (Davis et al., 2018), empathy (Pajevic et al., 2018), and other psychosocial factors.

The high intercorrelations between Machiavellianism, narcissism, and psychopathy—originally grouped as the Dark Triad—motivated the search for a *single* underlying disposition—Dark Factor of Personality (D) that could explain unethical, exploitative, and aggressive behavior (Miller et al., 2019). Moshagen et al. (2018) define D-factor as a general tendency to maximize one’s own utility while disregarding, accepting, or deliberately causing harm to others, supported by justifying beliefs and ideologies. Longitudinal research shows that D is highly stable over time, more so than individual dark traits, and that it prospectively shapes changes in those traits, consistent with the idea that D is a basic dispositional core from which specific dark profiles emerge (Zettler et al., 2020).

Individuals scoring high on Dark Triad traits tend to perform manipulative social strategies (Kajonius et al., 2015). Religious beliefs are negatively associated with psychopathy but positively with sadism (Schofield et al., 2021). Dark Triad traits can minimize life satisfaction and growth-oriented outcomes (Kaufman et al., 2019). High level of Machiavellianism is associated with hostile perceptions of others and limited interpersonal engagement (Rauthmann and Will, 2011). Machiavellianism and psychopathy—core expressions of D—show consistent negative links with intrinsic religiosity and positive with extrinsic religiosity (Czarna et al., 2025). Psychopathy has been shown to be negatively associated with the preservation of cultural, family, and religious traditions (Jonason et al., 2020), as well as general religiosity (Czarna et al., 2025). According to studies by Kammerle et al. (2014), religious and overall spiritual well-being are negatively correlated with subclinical psychopathy, whereas narcissism is positively related to certain aspects of religious well-being, such as hope and connectedness. Findings by Fekih-Romdhane et al. (2020) further indicate that existential well-being may serve as a predictor of psychopathy, accounting for up to 3.8% of the variance in regression models.

As Machiavellianism and psychopathy conflict with spiritual and general well-being (Liu et al., 2021; Blasco-Belled et al., 2023), narcissism is ambivalent: respondents who self-identify as non-religious tend to score lower in narcissism compared to religious individuals. Among the latter, narcissism shows a negative association with intrinsic religiosity but a positive association with extrinsic religiosity (Hermann and Fuller, 2017; Czarna et al., 2025). Agentic/grandiose narcissism can be positively related to certain religious or spiritual endorsements, but this appears driven by self-Enhancement motives (status, uniqueness, being “chosen”) rather than humility or compassion (Hermann and Fuller, 2017). Research suggests that, compared to the other Dark Tetrad traits, narcissism may be considered a relatively positive aspect, as it shows positive associations with extraversion, openness to experience, verbal intelligence, and tolerance for uncertainty (Krasavtseva and Kornilova, 2019).

As spiritual well-being reflects internalized meaning systems that emphasize empathy, responsibility, and mutuality, it is structurally incompatible with the exploitative, morally disengaged orientation of D (Peterman et al., 2002; Liaquat et al., 2013; Sun et al., 2015; Song and Park, 2020). On the other hand, the Dark core includes a tendency to use ideologies and moral codes instrumentally, spiritual and religious frameworks can be co-opted to justify superiority, control, or aggression. This leads to “dark” forms of spirituality—spiritual narcissism, spiritual entitlement, or punitive moralism (Hilbig et al., 2022). Skobrtal and Pospíšil (2023) demonstrated that these traits may, to a limited extent, predict existential satisfaction, accounting for 6.4% of variance.

A review of the existing literature indicates that empirical data on the relationship between spiritual well-being and malevolent personality traits of the Dark Tetrad remain limited and inconsistent. No such studies have yet been conducted in Russian-speaking samples, which underscores the novelty of the present research. The objective of this study is to examine the relationship between spiritual well-being and Dark Tetrad traits in a Russian-speaking population. The study hypothesizes that spiritual well-being will be negatively associated with Machiavellianism, psychopathy, narcissism, and sadism.

## 2 Materials and methods

### 2.1 Sample and procedure

The study of the relationship between spiritual well-being and Dark personality traits was conducted online in 2024. Invitations to participate were disseminated through student groups, Orthodox Christian communities, and interest-based groups on the social networks. Participants completed the study on an anonymous and voluntary basis without any compensation. After providing informed consent and demographic information, participants were asked to complete the two questionnaires. The final sample consisted of 959 respondents: 761 women (79.4%) and 198 men (20.6%), with a mean age of 38.6 ± 14 years. Among the participants, 341 (35.6%) were unmarried, 500 (52.1%) married, and 118 (12.3%) divorced; 577 individuals (60.2%) had children. A total of 129 respondents (13.5%) had completed secondary education, 203 (21.2%) secondary vocational education, 45 (4.7%) had incomplete higher education, and 582 (60.7%) held higher education degrees. The majority of respondents identified as Christians (*n* = 839, 87.5%) that reflects the general tendency for central Russia (about 79% of population are Christians in this region), as Muslims (*n* = 4, 0.4%), adherents of Slavic native faith (*n* = 2, 0.2%), atheists (*n* = 41, 4.3%), those who believed in God but did not associate themselves with any particular religion (*n* = 58, 6.0%), and other affiliations (*n* = 15, 1.6%).

### 2.2 Measures

Spiritual well-being was assessed using the *Spiritual Well-Being Scale* [SWBS; Paloutzian and Ellison (1982), adapted by Bakushkin and Ershova (2025)]. The scale consists of 20 items rated on a 6-point Likert scale (from 1 = strongly agree to 6 = strongly disagree) and includes two subscales: the Religious Well-Being Scale (maximum score = 60), which reflects well-being associated with one’s relationship with God (the transcendent dimension), and the Existential Well-Being Scale (maximum score = 60), which measures immanent well-being related to life satisfaction and sense of purpose.

Personality traits—Machiavellianism, subclinical narcissism, psychopathy, and sadism—were assessed using the *Short Dark Tetrad Questionnaire* (SD4; Paulhus et al., 2021), adapted by Kornienko et al. (2022). The questionnaire consists of 28 items rated on a 5-point Likert scale (from1 = strongly disagree to 5 = strongly agree) and includes four subscales: Machiavellianism, narcissism, psychopathy, and sadism, each comprising seven items (scores range: 7–35). Machiavellianism is defined by a strategic orientation toward interpersonal manipulation, emotional detachment, cynicism, and diminished empathy (Jones and Paulhus, 2009). Narcissism manifests as grandiosity, a pervasive sense of entitlement, and a readiness to exploit others for personal gain (Paulhus and Williams, 2002). Psychopathy is characterized by high impulsivity, callousness, low anxiety, and a propensity for deceptive behavior. Everyday sadism represents a normative disposition toward deriving pleasure from cruelty, encompassing tendencies to humiliate, dominate, and inflict physical or psychological suffering for gratification (Atadzhykova and Enikolopov, 2021), which is further linked to emotional coldness and deficient empathy (Nell, 2006; Pajevic et al., 2018).

### 2.3 Statistical analytical plan

This study employed a cross-sectional observational design. All statistical computations and data visualization were performed in the R environment for statistical computing, version 4.5.1 (R Foundation for Statistical Computing). Descriptive statistics are presented as absolute and relative frequencies for categorical variables, as mean (±standard deviation) and median (1st and 3rd quartiles) for continuous variables with symmetric distribution, and as median (1st and 3rd quartiles) otherwise. Bivariate associations between indicators of spiritual well-being and the traits of the Dark Tetrad were assessed using Spearman’s rank-order correlation coefficient (*ρ*), with 95% confidence intervals (CI) reported for each estimate. To preliminarily evaluate predictive relationships, univariate linear regression analysis was performed. Subsequently, a multivariate linear regression model was constructed to identify the most robust set of predictors. Predictor variables were selected via a bidirectional stepwise algorithm based on adjusted *R*^2^. The initial model for the stepwise procedure was the univariate model demonstrating the highest explanatory power (*R*^2^). Key assumptions of the final linear regression model, including homoscedasticity, independence of residuals, and absence of multicollinearity (assessed via Variance Inflation Factors, VIF < 5), were verified and met.

## 3 Results

Table 1 presents the results of the Spiritual Well-Being Scale (SWBS) and Short Dark Tetrad Questionnaire in test scores. The results reflect a moderate level of spiritual well-being in most respondents. The highest values of “Dark traits” were observed for the *Machiavellianism* scale, and the lowest for the *Sadism* scale.

**Table 1:** Scores on spiritual well-being scale and Dark Tetrad traits.

Components	M (±SD)	Me (Q1–Q3)
Spiritual Well-Being Scale (SWBS)*	96.8 (±16.7)	100 (89–109)
Religious Well-Being Scale (RWBS)*	49.8 (±11.9)	54 (47–58)
Existential Well-Being Scale (EWBS)***	47 (±8.1)	48 (42–53)
Machiavellianism	19.2 (±4.7)	19 (16–22)
Narcissism	16.9 (±5.4)	17 (13–20)
Psychopathy	14.5 (±4.5)	14 (11–17)
Sadism	12.4 (±4.8)	11 (9–15)

*Scores ranging from 20 to 40 indicate low overall spiritual well-being; 41–99 indicate moderate spiritual well-being; and 100–120 indicate high spiritual well-being. **Scores ranging from 10 to 20 reflect dissatisfaction in one’s relationship with God; 21–49 indicate moderate religious well-being; and 50–60 reflect a positive perception of one’s relationship with God. *** Scores ranging from 10 to 20 indicate low life satisfaction and a possible lack of clarity regarding life purpose; 21–49 suggest a moderate level of satisfaction and presence of life goals; and 50–60 reflect high life satisfaction and a clear sense of purpose.

The results of the correlation analysis of spiritual well-being indicators and Dark Tetrad traits are presented in Table 2.

**Table 2:** Correlations between spiritual well-being and Dark Tetrad traits (*N* = 959).

“Dark” Traits	Spiritual Well-Being Scale, SWBS		
The Short Dark Tetrad Questionnaire (SD4)	Religious Well-Being Scale, RWBS	Existential Well-Being Scale, EWBS	Spiritual Well-Being Scale, SWBS
Machiavellianism	−0.25 [−0.31; −0.19] *p* < 0.001	−0.22 [−0.28; −0.16] *p* < 0.001	−0.29 [−0.34; −0.23] *p* < 0.001
Narcissism	−0.21 [−0.26; −0.14] *p* < 0.001	0.04 [−0.03; 0.1] *p* = 0.277	−0.12 [−0.18; −0.06] *p* < 0.001
Psychopathy	−0.14 [−0.2; −0.08] *p* < 0.001	−0.24 [−0.3; −0.18] *p* < 0.001	−0.22 [−0.28; −0.16] *p* < 0.001
Sadism	−0.37 [−0.43; −0.32] *p* < 0.001	−0.23 [−0.29; −0.17] *p* < 0.001	−0.38 [−0.43; −0.32] *p* < 0.001

Correlation analysis revealed a distinct pattern between the facets of spiritual well-being and the Dark Tetrad. Both Religious Well-Being (RWB) and the Spiritual Well-Being (SWB) score were inversely associated with Machiavellianism (*p* < 0.001), Psychopathy (*p* < 0.001), Sadism (*p* < 0.001), and Narcissism (*p* < 0.001). Existential Well-Being (EWB), demonstrated significant negative correlations with Machiavellianism (*p* < 0.001), Psychopathy (*p* < 0.001), and Sadism (*p* < 0.001), showing no significant link to Narcissism.

The predictive utility of the Dark Tetrad traits for religious, existential, and spiritual well-being was examined through hierarchical regression. Initial univariate models (Table 3) demonstrated that all traits were significant individual negative predictors of Religious Well-Being (RWB) at *p* < 0.001. Specifically, Sadism showed the largest effect size (*β* = −1.23, 95% CI [−1.37, −1.09]), accounting for 24.5% of the variance (*R*^2^ = 0.245). The coefficients for the remaining traits were as follows: Machiavellianism (*β* = −0.68, 95% CI [−0.83, −0.52]), Narcissism (*β* = −0.49, 95% CI [−0.63, −0.36]), and Psychopathy (*β* = −0.44, 95% CI [−0.61, −0.28]).

**Table 3:** Univariate regression of Dark Tetrad traits on Religious Well-Being (RWB) subscale.

Components	*β*	95% CI	*p*	*R* ^2^
Sadism	−1.23	−1.37; −1.09	<0.001	24.5%
Machiavellianism	−0.68	−0.83; −0.52	<0.001	7.2%
Narcissism	−0.49	−0.63; −0.36	<0.001	5%
Psychopathy	−0.44	−0.61; −0.28	<0.001	2.9%

Table 4 presents the results of a multiple regression analysis performed to identify predictors of Religious Well-Being, using the adjusted coefficient of determination for model comparison. The baseline (null) model included the Sadism scale as a sole predictor. The introduction of Psychopathy as a second independent predictor increased the proportion of explained variance in the dependent variable (Religious Well-Being) from 24.5 to 26.0%. The contribution of each predictor was assessed independently, as no evidence of multicollinearity was found in the model (Variance Inflation Factor, VIF = 1.43). Notably, compared to the univariate analysis, the direction of the association for the Psychopathy factor reversed in the multivariate model, revealing a significant positive correlation (*β* = 0.38, 95% CI [0.21, 0.55], *p* < 0.001).

**Table 4:** Multiple regression analysis of the Dark Tetrad components as predictors of the Religious Well-Being (RWB) subscale.

Components	*β*	95% CI	*p*	VIF
Intercept	62	–	–	–
Sadism	−1.43	−1.59; −1.27	<0.001	1.43
Psychopathy	0.38	0.21; 0.55	<0.001	1.43

Scores on the *Existential Well-Being (EWB)* scale (Table 5) were significantly associated (*p* < 0.001) with the factors *Psychopathy* (*β* = −0.43 [95% CI: −0.54; −0.32]), *Sadism* (*β* = −0.38 [95% CI: −0.49; −0.28]), and *Machiavellianism* (*β* = −0.39 [95% CI: −0.49; −0.28]). Among these, the strongest predictive power was demonstrated by the *Psychopathy* factor, which explained 6% of the variance.

**Table 5:** Univariate analysis of the Dark Tetrad components as predictors of spiritual well-being.

Components	*β*	95% CI	*p*	*R* ^2^
Sadism	−1.62	−1.81; −1.42	<0.001	21.3%
Machiavellianism	−1.07	−1.28; −0.85	<0.001	9.1%
Psychopathy	−0.88	−1.1; −0.65	<0.001	5.7%
Narcissism	−0.44	−0.63; −0.24	<0.001	2%

As shown in Table 6, scores on the Existential Well-Being (EWB) scale were significantly associated with three predictors: Psychopathy (*β* = −0.43, 95% CI [−0.54, −0.32]), Sadism (*β* = −0.38, 95% CI [−0.49, −0.28]), and Machiavellianism (*β* = −0.39, 95% CI [−0.49, −0.28]), all *p* < 0.001. Of these, the Psychopathy factor demonstrated the strongest predictive power, uniquely accounting for approximately 6% of the variance in EWB scores (*R*^2^ = 0.06).

**Table 6:** The univariate regression analysis of the Dark Tetrad components as predictors of Existential Well-Being Scale (EWBS).

Components	*β*	95% CI	*p*	*R* ^2^
Psychopathy	−0.43	−0.54; −0.32	<0.001	6%
Sadism	−0.38	−0.49; −0.28	<0.001	5.2%
Machiavellianism	−0.39	−0.49; −0.28	<0.001	5.2%
Narcissism	0.06	−0.04; 0.15	0.25	0.1%

Table 7 presents the results of a multiple regression analysis. A baseline model containing only Psychopathy scale scores was first established. The subsequent inclusion of Machiavellianism, Narcissism, and Sadism as additional predictors significantly improved the model, increasing the total explained variance to 12.2% (*R*^2^ = 0.122). In this multivariate context, a statistically significant positive association emerged for Narcissism, which was not evident in the univariate analyses (*β* = 0.32, 95% CI [0.22, 0.42], *p* < 0.001).

**Table 7:** Multiple regression analysis of the Dark Tetrad components as predictors of Existential Well-Being Scale (EWBS).

Components	*β*	95% CI	*p*	VIF
Intercept	55	–	–	–
Psychopathy	−0.31	−0.44; −0.18	<0.001	1.46
Sadism	−0.22	−0.35; −0.08	0.002	1.8
Machiavellianism	−0.31	−0.43; −0.19	<0.001	1.42
Narcissism	0.32	0.22; 0.42	<0.001	1.28

Univariate regression analyses, presented in Table 5, indicated that scores on the Spiritual Well-Being (SWB) scale were significantly associated with all four traits of the Dark Tetrad (*p* < 0.001 for all). The standardized coefficients (*β*) were as follows: Sadism (*β* = −1.62, 95% CI [−1.81, −1.42]), Machiavellianism (*β* = −1.07, 95% CI [−1.28, −0.85]), Narcissism (*β* = −0.88, 95% CI [−1.10, −0.65]), and Psychopathy (*β* = −0.44, 95% CI [−0.63, −0.24]). The Sadism scale was the strongest predictor, uniquely explaining 21.3% of the variance in SWB scores (*R*^2^ = 0.213).

Table 8 presents the results of a hierarchical multiple regression analysis. A baseline model containing only the Sadism scale was first established. The subsequent addition of Machiavellianism and Narcissism as predictors resulted in a final model explaining 22.5% of the variance in the dependent variable (*R*^2^ = 0.225). Notably, a significant change was observed for Narcissism: while its association was negative in the univariate analysis, it became a significant positive predictor in the multivariate model (*β* = 0.26, 95% CI [0.07, 0.46], *p* = 0.009), suggesting a potential suppressor effect.

**Table 8:** Results of the multiple regression analysis of the Dark Tetrad components as predictors of scores on the Spiritual Well-Being Scale (SWBS).

Components	*β*	95% CI	*p*	VIF
Intercept	119	–	–	–
Sadism	−1.55	−1.79; −1.31	<0.001	1.47
Machiavellianism	−0.38	−0.62; −0.15	0,002	1.41
Narcissism	0.26	0.07; 0.46	0.009	1.27

## 4 Discussion

This study examined the relationship between spiritual well-being—an integrative indicator of health and life satisfaction—and “Dark Personality” traits: Machiavellianism, psychopathy, narcissism, and sadism. Across all analyses, the most consistent pattern is that higher levels of the dark traits are associated with lower religious, existential, and overall spiritual well-being (Neumann et al., 2021) and present data supported this pattern. As it was shown below, this effect is best understood through the lens of a common Dark Factor (D-core) that reflects a tendency toward callous self-interest, willingness to harm others, and the rationalization of such harm as acceptable or deserved (Dinic et al., 2024). In the present study, *Sadism* emerges as the clearest behavioral manifestation of the D-core. It shows the strongest negative correlations with spiritual well-being and the largest unique predictive effects across religious and overall spiritual outcomes. This is consistent with behavioral studies demonstrating that everyday sadists will actively work for the opportunity to inflict suffering, even when it is costly and unprovoked, underlining a genuine “appetite for cruelty” rather than mere impulsivity or instrumental aggression (Buckels et al., 2013). The fact that *Sadism* alone explains over one fifth of the variance in spiritual well-being suggests that the most malevolent expression of the D-core—pleasure in others’ pain—is fundamentally irreconcilable with the compassion, benevolence, and transcendence that characterize spiritual health. Converging evidence from applied contexts shows that sadism is also a robust predictor of harmful interpersonal behavior in everyday life, such as severe counterproductive work behavior, further underscoring its destructive core (Fernandez-del-Rio et al., 2022).

Psychopathy and Machiavellianism also index the D-core, but with a somewhat broader and more strategic profile. Their zero-order negative effects on religious and existential well-being indicate that impulsivity, emotional coldness, and manipulative exploitation erode both vertical (God-related) and horizontal (meaning- and purpose-related) aspects of spirituality. This aligns with findings that these traits promote aggressive, antisocial, and object-related destructive behaviors, including acts such as fire setting and other forms of object-directed violence (Wehner et al., 2022).

The regression models help disentangle what is core and what is residual within each trait. When the shared D-core is statistically partialled out—primarily through the inclusion of Sadism, Psychopathy, and Machiavellianism—Narcissism shifts from a null or weakly positive bivariate relation with existential and spiritual well-being to a clear positive predictor, which is consistent with previous research (Czarna et al., 2025; Wink and Dillon, 2003). This pattern is consistent with broader evidence that, when separated from pronounced antagonism and callousness, narcissism can be associated with higher well-being, via more optimistic and agentic conceptions of happiness (Kammerle et al., 2014; Krasavtseva and Kornilova, 2019; Joshanloo, 2021; Skobrtal and Pospíšil, 2023). In the current results, the “dark” part of narcissism appears to belong to the D-core and thus harms spiritual outcomes, whereas the remaining components (self-confidence, a stable sense of worth, and perceived personal significance) can support both existential well-being and the construction of a coherent spiritual self-narrative.

The D-core lens clarifies why spiritual well-being is particularly sensitive to sadistic and psychopathic tendencies under conditions of existential threat. Experimental work shows that individuals high in everyday sadism are especially prone to destructive, dominance-oriented behavior when their self is existentially threatened, using aggression to restore a sense of control and self-esteem (Buckels et al., 2013; Pfattheicher and Schindler, 2015; Fernandez-del-Rio et al., 2022). By contrast, spiritually well individuals typically respond to existential anxiety by deepening meaning, connection, and creative engagement with life (David and Truța, 2023). Thus, when the D-core is strong, existential threat is more likely to trigger domination and cruelty than growth-oriented meaning-making, directly undermining both existential and spiritual well-being. Conversely, when sadistic traits are isolated, core psychopathic features such as callousness and low anxiety can facilitate access to transcendental support (Fekih-Romdhane et al., 2020; Schofield et al., 2021), thereby potentially enhancing spiritual well-being.

Taken together, the pattern of correlations and regressions indicates that spiritual well-being is not merely inversely related to individual dark traits, but is systematically shaped by the underlying D-core. Traits that are heavily saturated with this core (especially Sadism, but also Psychopathy and Machiavellianism) consistently damage religious, existential, and overall spiritual functioning. Traits that blend D-core and non–D-core components (notably Narcissism) only show their potentially adaptive side when the common malevolent variance is controlled.

In this sense, spiritual well-being appears to be especially “core-sensitive”: it is diminished by any personality configuration organized around callous self-interest and the justification of harm, yet it can integrate and even benefit from agentic, self-assured qualities once they are decoupled from this dark motivational nucleus.

## 5 Limitation

Several limitations of this study should be noted. First, the cross-sectional design and the use of self-report measures preclude causal inferences and may be susceptible to common methodological biases (e.g., social desirability). Second, while the use of a bidirectional stepwise algorithm for building multiple regression models enhanced their predictive power, it also increases the risk of overfitting, thus, the models require replication with independent samples. Third, the significant gender imbalance (predominantly female sample) limits generalizability, as expressions of “Dark Tetrad” traits may be gender-specific. Future studies should either recruit gender-balanced cohorts or conduct stratified analyses. Finally, the identified reversals in the direction of relationships (e.g., for psychopathy and narcissism when accounting for the common D-core) offer intriguing but speculative insights into suppressor effects. They generate important hypotheses about the distinct functional role of each trait but must be substantiated by future theoretical and empirical work employing longitudinal or experimental methods.

## Data Availability

The materials, data, and syntax for this study are available at https://doi.org/10.6084/m9.figshare.30223333.
